# An ERP Study on the Role of Phonological Processing in Reading Two-Character Compound Chinese Words of High and Low Frequency

**DOI:** 10.3389/fpsyg.2021.637238

**Published:** 2021-02-25

**Authors:** Yuling Wang, Minghu Jiang, Yunlong Huang, Peijun Qiu

**Affiliations:** ^1^Center for Psychology and Cognitive Science, Tsinghua University, Beijing, China; ^2^Advanced Innovation Center for Future Education, Beijing Normal University, Beijing, China; ^3^Laboratory of Cognitive Linguistics, Department of Foreign Languages and Literature, Tsinghua University, Beijing, China; ^4^College of International Sport Organizations, Beijing Sport University, Beijing, China

**Keywords:** Chinese word recognition, homophonic, N400, P200, word frequency

## Abstract

Unlike in English, the role of phonology in word recognition in Chinese is unclear. In this event-related potential experiment, we investigated the role of phonology in reading both high- and low-frequency two-character compound Chinese words. Participants executed semantic and homophone judgment tasks of the same precede-target pairs. Each pair of either high- or low-frequency words were either unrelated (control condition) or related semantically or phonologically (homophones). The induced P200 component was greater for low- than for high-frequency word-pairs both in semantic and phonological tasks. Homophones in the semantic judgment task and semantically-related words in the phonology task both elicited a smaller N400 than the control condition, word frequency-independently. However, for low-frequency words in the phonological judgment task, it was found that the semantically related pairs released a significantly larger P200 than the control condition. Thus, the semantic activation of both high- and low-frequency words may be no later than phonological activation.

## Introduction

One critical issue for any model of visual word recognition and reading is the specification of the role of phonology during lexical access (Carreiras et al., 2014). Phonological information is necessary if the word is to be read aloud; however, it is not clear whether phonology is activated during silent reading. At present, most research into the role of phonology in reading has focused on alphabetic languages and have explored this primarily through behavioral and event-related potential (ERP) experiments. Many findings were consistent with the view that phonology is activated early and used for semantic access, as seen in English (Pollatsek et al., 1992; Grainger et al., 2006; Ashby and Martin, 2008; Ashby, 2010; Wilson et al., 2011); Hebrew (Frost et al., 2003); French (Ferrand and Grainger, 1992, 1994); and Spanish (Pollatsek et al., 2005; Carreiras et al., 2009).

To examine the influence of phonological effects during lexical access, most ERP studies have applied priming procedures. In alphabetic language, there is usually a strong spelling-sound mapping. Thus, the orthographic difference was always confounded with a phonological difference. In order to verify the phonological effects, orthographic variables need to be strictly controlled. For example, in the experiment of Carreiras et al. (2009), two sets of priming words similar in orthography to the target words were set to verify the priming effect of phonology. In other words, the difference between the priming words and the target words under conditions of critical phonological comparison was only manifested in the phonology, so if the priming effect occurs, it can be attributed to the role of phonology. Similarly, the difference between the priming words and the target words under the critical orthographic conditions was only manifested in the orthographic factors. Orthographic priming was mainly obtained in the 150–250-ms (N250) time window, while phonological priming occurred in the 350–550-ms (N400) window. Thus, These results strongly suggest that there is a phonological priming effect, which cannot be attributed to uncontrolled orthographic factors.

It is not difficult to understand that phonology plays an important role in lexical access of alphabetic language, as such languages usually have a strong spelling-sound mapping. Does phonology play an important role in all writing systems? To solve this question, researchers need to include languages with weak print-to-sound mapping. Compared with English, Chinese is a logographic script whose characters represent morphemes rather than phonemes (Yu and Reichle, 2017). Moreover, there are a large number of homophones in Chinese: a specified syllable (e.g., /qing1/) contains many different shapes of characters with distinct meanings (e.g., 清，倾，轻，卿，氢，顷). This linguistic feature gives us the opportunity to study the effect of phonology without orthographic confusion. Earlier papers on reading Chinese words have interpreted the role of phonology differently, largely based on behavioral data. For example, some scholars claimed that phonology plays an important role when reading Chinese characters or words (Perfetti and Zhang, 1995; Tan and Perfetti, 1997; Xu et al., 1999; Spinks et al., 2000; Guo et al., 2005). Others (Zhou and Marslen-Wilson, 1999, 2000; Zhou et al., 1999; Chen and Shu, 2001), however, held the opposite view. Additionally, in recent years, cognitive neuroscience has rekindled this debate through the introduction of techniques, such as ERP monitoring which has the appropriate temporal resolution to track the time-course of processing (Carreiras et al., 2014). Nevertheless, current ERP studies on the importance of phonology have not yet reached a consensus result regarding Chinese reading. Research on Chinese word recognition was currently based on the assumption that orthographic information is perceived in a feed-forward manner, and then other representations such as phonology or semantics were activated. Based on this feedforward activation method, the controversy focuses on whether the phonology is activated before the semantics, that is, whether the phonology acts as an information transmission bridge between orthographic and semantic information. Thus, 3 different assumptions about the recognition of Chinese words have been proposed, namely the direct access view (Wang et al., 2010; Wang, 2011), the phonological mediation view (Liu et al., 2003; Ren et al., 2009), and the dual-route view (Meng et al., 2008; Zhang et al., 2009; Liu et al., 2011).

Why are the ERP results inconsistent for Chinese word recognition? After comparing most ERP studies that explore the effect of Chinese, we found that most studies have different experimental paradigms, mainly including the priming paradigm and the violation paradigm. In the violation paradigm, the critical word is typically at the end of a sentence. In addition, the experimental materials are also different, and mainly divided into Chinese single-character words and two-character compounds. Finally, with regard to the properties of experimental materials, such as frequency factors, some studies take into account, while others do not. Below we will compare these related studies in detail to find the best way to solve the problem.

First, most experiments essentially focused on Chinese single-character words. For example, using color words, homophones of color words, and color word-associates as materials in a Stroop task, in which participants were asked to respond to the ink or back-color of words and to ignore the word meaning, Wang et al. (2010) found an N450 component for incongruent color words (“red” printed in green ink) and color word-associates (“fire” printed in green ink). If a word could activate the meaning of other words with the same pronunciation, an N450 component would have been expected for incongruent homophones. However, there was no difference in the ERP waveforms elicited by congruent (“洪”, /hong2/, meaning flood, homophone of “red” in Chinese, printed in red ink) and incongruent (“洪” printed in green ink) homophones in the N450 time-window, indicating that phonology does not play an important role in Chinese semantic access. Then, they extended their work and added pseudowords that were orthographically similar to these color words, and an N450 was also observed in both incongruent conditions (Wang, 2011), which further proved that semantic access to Chinese one-character words may rely heavily on the orthography–semantic route.

However, Zhang et al. (2009) indicated that word frequency affects the time-course of phonological and semantic activation in Chinese single-character word recognition. Different from the above Stroop task, semantic and phonological judgment tasks were used to detect the order of phonological and semantic activation in word recognition. For the semantic judgment task, the behavioral and ERP response to target words preceded by homophones and irrelevant words can be compared to reveal when and whether phonology interferes with semantic access. Similarly, for the phonological judgment task, the behavioral and ERP response to target words preceded by semantically related and irrelevant words can be compared to reveal when and whether semantic interferes with phonological judgement. Then, the relative time course of phonological and semantic activation can be obtained by comparing the latency of related ERP components, and the influence of word frequency can also be tested according to the activation patterns. For example, if phonological activation interferes with semantic judgment, but semantics does not interfere with phonological judgment, then it can be inferred that phonological activation is earlier than semantics. The above reasoning is based on the assumption that earlier activation will affect the later component processing, but not vice versa (Perfetti and Zhang, 1995).

Specifically, Zhang et al. (2009) used unrelated or related semantical and phonological word-pairs of high and low frequency as materials in semantic judgment tasks and homophone judgment tasks. They found that a smaller N400 component (as compared to the unrelated control words) was elicited in response to both the homophonic pairs in the semantic task and the semantically related pairs in the homophone task of both high and low frequency, suggesting activation of both phonology and semantics in the N400 time-window. However, for low-frequency words only, the homophonic pairs in the semantic task evoked a larger P200 component, relative to control words, which implies early phonological processing. Therefore, their results indicated that, for low-frequency pairs, phonology activates earlier than semantics. For high-frequency words, N400 component from both phonological and semantic activation revealed no differences in their time-course. However, behavioral data revealed that semantics interferes with phonological processing and that there was no phonological priming effect on semantic processing, indicating that semantic information was activated earlier than phonology for high-frequency words. Thus, their results showed that phonology tends to be inactive during the semantic access of high-frequency words, while the opposite occurred for low-frequency words, indicating that word frequency affects the role of phonology in semantic access to Chinese single-character words (Zhang et al., 2009).

Using the same rationale of the interference paradigm in semantic and homophone judgment tasks, Liu et al. (2003) found that, in a meaning decision task, homophone pairs, but not semantic relatedness manipulation, led to a reduction in amplitude in the N400 time-window relative to unrelated control words in a pronunciation decision task. On the whole, the P200 indicating the priming effect of phonology was not found in their experiment. Nevertheless, in the N400 time-window, homophone interference effects were seen, but the reverse effect—meaning interference in pronunciation—was not observed. Thus, the results of Liu et al. (2003) supported that phonology activates earlier and plays an important role in semantic access of Chinese single-character words.

Here, we will summarize the above studies on the phonological and semantic activation of Chinese character recognition. First, the paradigms of these studies differed. It is worth noting that the Stroop task is similar to a naming task. The preferred method for exploring the role of phonology in word recognition is lexical decision tasks, rather than naming tasks, because naming tasks may have intrinsic phonetic components that are independent of lexical access (Carreiras et al., 2009). Furthermore, the attributes of the Stroop task determine that the experimental materials can only contain a few color words and their associates, which are not representative. Second, the word frequency factor was not taken into account in most earlier experiments, although frequency is the main factor affecting word recognition (Forster and Chambers, 1973; Frederiksen and Kroll, 1976; Balota et al., 2004). So far, the frequency of words in a certain language has the greatest impact on their recognition methods, and words with higher frequencies in the language are recognized faster (Norris, 2013). Last, in terms of experimental materials, separate pseudowords that are not included in the context of the sentence (Wang, 2011), may not activate semantic representations strongly (Meng et al., 2008). Thus, in this study, we did not use separate pseudowords as experimental material.

The remaining ERP research on the order of phonology and semantic processing in Chinese single-character word reading was mostly carried out in sentences, and the conclusions were inconsistent (Meng et al., 2008; Ren et al., 2009; Liu et al., 2011). In terms of linguistic brain mechanisms, a complete sentence is rather unmanageable, whereas a short two-word phrase is a more manageable representation unit (Pylkkänen, 2019). If the task of word recognition was explored at the end of sentence, more uncontrollable variables, such as syntax, semantic context, word position, and other factors, could potentially lead to inconsistent conclusions (Halgren et al., 2002).

However, to our knowledge, there has been only one ERP study on the phonological effect of Chinese two-character compound words. Wong et al. (2014) used the masked priming paradigm to ask participants to determine whether each target word is a legal compound in Chinese. Specifically, the priming word and the target word share a same character under the character-related conditions, a syllable under the syllable-related conditions, related or similar semantic under semantic-related conditions, and irrelevant under the control condition. Compared with the control condition, a significant reduction in ERP amplitudes was observed in the character-related condition in the time-window of 150–250 ms (N250) post target. In addition, in the semantic-related condition, the attenuation in ERP amplitude was found in the window of 250–500 ms (N400). It is worth noting that no significant effects were found in syllable-related conditions. Consequently, phonology plays a subsidiary role in reading Chinese two-character compound words, according to that investigation (Wong et al., 2014).

One potential limitation of the study of Wong et al. (2014) is that word frequency was not taken into account; thus, their conclusion may not represent all Chinese two-character compound words. Secondly, in order to verify the priming effect of phonology, the experiment only set the homophone of the first constituent character or the second character of two-character compound words. In other words, the experiment did not set a whole word of homophone-priming that was identical to the phonology of the target word. Therefore, a word-level phonological priming effect of Chinese two-character compound words cannot be completely excluded. More importantly, the prime and target shared an identical constituent character in the character-related condition, indicating that the character-related primes and their targets shared identical orthographic and phonological contents. Thus, it remains unknown how much phonology contributes to the N250 associated with wordform processing. In addition, the effects observed in the character-related condition might be due to morphological priming since the character-related primes and the targets shared similar constituent morpho-orthographic features. Based on the above-mentioned reports, in this study, we aimed to explore the role of phonology in reading of both high- and low-frequency Chinese two-character compound words. We set out to use a more rigorous experimental design, based on the study of Wong et al. (2014). However, dissimilar to the paradigm of Wong et al. (2014), meaning and pronunciation judgment tasks were set in our study similar to some earlier studies (Tan and Perfetti, 1999; Liu et al., 2003; Zhang et al., 2009). In other words, in our paper, the interference paradigm of exploring a single-character word was first adopted to explore the role of phonology in reading Chinese two-character compound words. This paradigm of word recognition could explore the semantic route more accurately and comprehensively, as it involves not only the conventional semantic judgment task, but also the phonological task. The semantic task could monitor whether and when phonology interferes with semantic access. Similarly, the phonological judgment task could monitor whether and when semantics interferes with phonological processing. Therefore, the final conclusion about the relative time-course of phonological and semantic activation in word recognition would not be derived from a single task, but from repeated verification of 2 tasks. Moreover, both high- and low-frequency words would be verified separately. We also designed a priming paradigm of 3 types for phonologically related, i.e., homophones, semantically related, and unrelated pairs.

In our paper, 2 ERP components were monitored: P200 and N400, which are implicated in phonological and semantic processing and have been studied extensively and which form the basis of a rich of literature, particularly in Zhang et al. (2009). We would monitor whether a reduced N400 component induced by phonologically related pairs in semantic tasks and semantically related pairs in phonological tasks, indicating that both semantic and phonology may activate in the N400 time window. More importantly, we would examine whether homophone pairs have the P200 component that characterizes early phonological activation in the semantic judgment task, and whether this effect is different in high and low frequency words. In short, whether semantics and phonology have an obvious activation sequence will be verified through 2 interference tasks.

## Methods

### Participants

Twenty-four (mean age = 21.9 years, range = 19–26 years, 14 males) native Chinese students from Tsinghua University were paid to participate in this experiment. All of them were right-handed according to the Edinburgh handedness test (Oldfield, 1971), and all reported normal or corrected-to-normal vision before the experiment. None of them reported any history of neurological or psychiatric impairment. Informed consent was obtained from all participants, in accordance with the Helsinki Declaration. This study adheres to the ethical procedures for the protection of human participants in research and was approved by the ethics committee of the School of Psychology at Tsinghua University.

### Materials and Design

The target words consisted of 120 Chinese two-character compounds, and both high-and low-frequency words accounted for half the words. Each of the targets was paired with 3 analogs: a phonologically identical word (a homophone), a word of related meaning, and a control (unrelated) word. High-frequency target words were always paired with high-frequency preceding words, and both tended to be consistent in terms of the number of strokes and frequency. In addition, the semantic association between semantically related pairs were strictly controlled. A separate group of 30 participants evaluated the degree of semantic relevance between the semantically related pairs used in this study on a 7-point scale, with 1 reflecting the lowest and 7 the highest relevance. The average score of the high-frequency semantically related word-pairs was 5.62, and the average score of the low-frequency pairs was 5.73, with no significant difference between these (*p* > .1). In addition, the semantic relatedness between the homophonic pairs and unrelated pairs was also no significant difference (*p* > .1). All stimuli used in this study are shown in Appendix.

All materials in this experiment are selected from the Xiandai Hanyu Pinlu Cidian [Modern Chinese Frequency Dictionary] (1986). The low-frequency words are selected from a word list of less frequently used words in the dictionary which every word appears <8 times per million. The high-frequency words in this experiment are mainly selected from the high-frequency list in the dictionary which every word appears more than 800 times per million.

The whole experiment was divided into 6 blocks, three for semantic judgment tasks and three for phonological judgment tasks. The order of item presentation within each block was random and the block order was counter-balanced across-subjects. There were 144 trials per block, including 120 test trials and 24 filler trials. Half of the trials used high-frequency word-pairs and the other half used low-frequency word-pairs. For each frequency, 20 trials were performed on semantically related pairs, 20 trials were performed on homophone pairs, and 20 trials were performed on unrelated pairs. Consequently, a total of 864 trials were included in the present experiment (Zhang et al., 2009). Each target word appears only once in each block. Filler trials were used to reduce reaction bias due to unequal number of trials requiring positive or negative reactions.

### Procedure

Participants were seated comfortably in a sound-attenuated, dimly lit room. They were instructed to focus their eyes on the middle of the screen and avoid any body movements, but to relax during the experiment. All the stimuli were presented word by word on an LCD computer screen located 1-m away from the participants. Each core structure began with a “+” sign that lasted for 300 ms, and then the preceding word was presented for 140 ms, with no interval (Zhang et al., 2009). Thereafter, a blank screen was presented for 360 ms, followed by the target word presented for 500 ms. Then, a questioning cue “?” was presented until the participant were asked to press a button as quickly and accurately as possible once they made their decision for the word-pairs just presented. For semantic tasks, participants needed to judge whether the word-pair was semantically related. For phonology tasks, subjects needed to judge whether the two words were homophones.

Before the formal test, each participant executed 60 practice trials. If the accuracy in the practice module reached 80%, the formal test started. Participants could take a 2–5-min break between blocks. The experiment, including electrode preparation, lasted about 1.5–2 h.

### ERP Recordings and Analyses

Electroencephalogram (EEG) were recorded from 62 Ag/AgCl electrodes in an elastic cap configured with an international 10–20 electrode placement system (Easycap; Brain Products GmbH, Gilching, Germany). The vertical electro-oculogram (VEOG) was recorded by the electrodes below the left eye, and the horizontal electro-oculogram (HEOG) was recorded by the electrodes at the outer canthus of the right eye. All electrode impedances were kept below 10 kΩ. The EEG signal was amplified by the BrainAmpDC amplifier system (Brain Products GmbH) with a bandpass of 0.01–100 Hz and was continuously sampled at 500 Hz (Huang et al., 2018).

**Figure 1:**
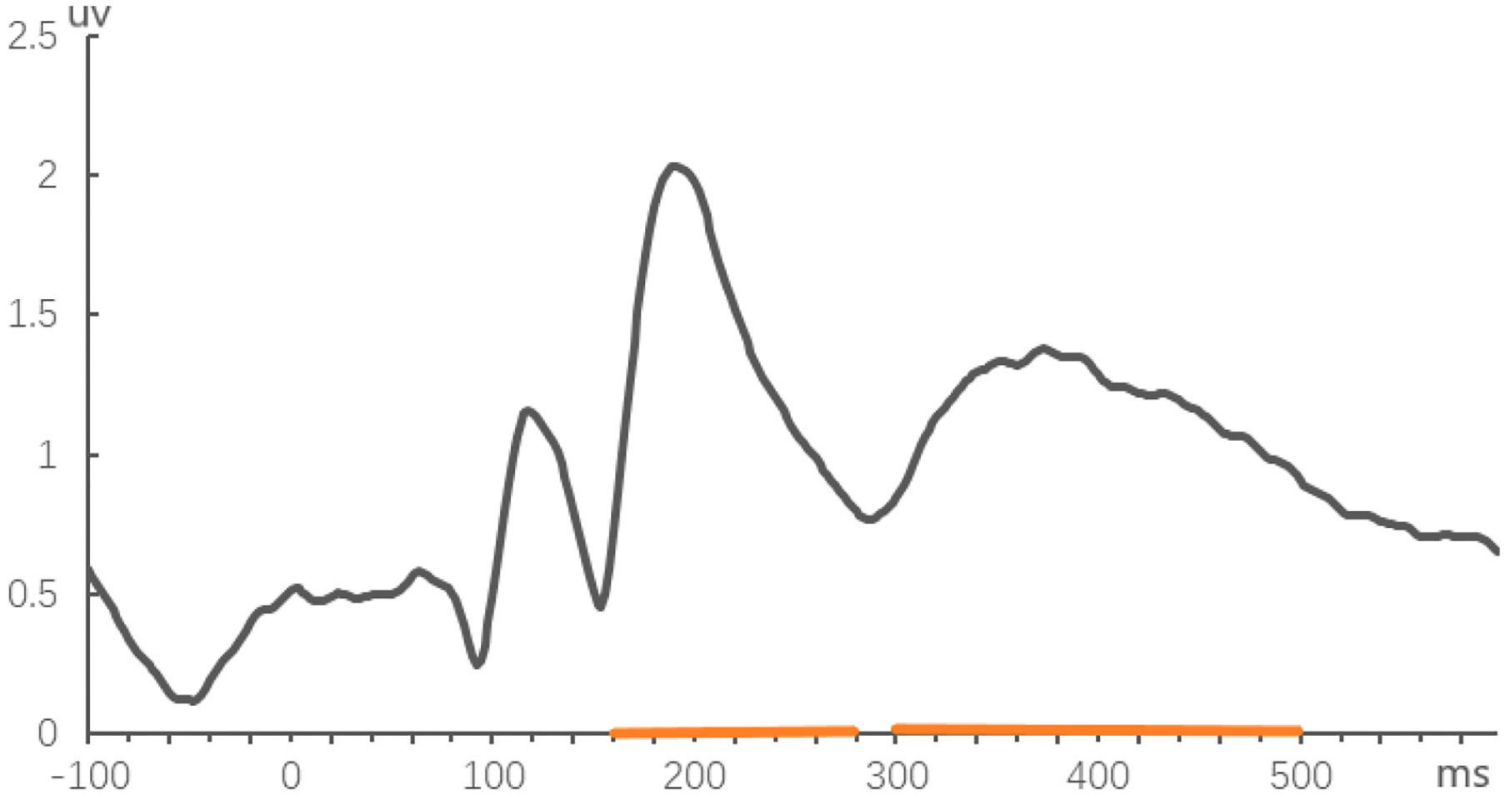
Global field power averaged across all experimental conditions and across 24 subjects.

The ERPs were computed from the 100 ms before to 600 ms after the onset of the target word (100 ms pre-target baseline). Semi-automatic Ocular Correction with Independent Component Analysis was adopted. The EEGs were bandpass-filtered offline from 0.05 to 30 Hz (zero phase shift mode, 24 dB/oct). Epochs exceeding ±80 μV were automatically discarded by artifact rejection and trials that responded incorrectly were also eliminated. Overall, 17.4% of the trials were discarded. Two time-windows were selected on the basis of Global Field Power (GFP) (Zhang et al., 2019; see Figure 1): at 160–280 ms and 300–500 ms for P200 and N400 components, respectively. According to the wide distribution of N400 and P200 in topographic maps, ERPs were analyzed separately for midline and lateral electrodes. Repeated measures analysis of variance (ANOVA) was performed on the average amplitude of each time window. The three within-participant factors involved were frequency (high and low), relationship type (semantically or phonologically related, or unrelated), and lateral areas or midline electrodes (Fz, Cz, and Pz). The laterality and anteriority factors were crossed, forming 6 lateral regions of interest (ROI) (Carreiras et al., 2009; Wong et al., 2014; Wu et al., 2017), and each region has 6 representative electrodes: left anterior/LA (F3, F5, F7, FC3, FC5, and FT7); left central/LC (C3, C5, T7, CP3, CP5, and TP7); left posterior/LP (P3, P5, P7, PO3, PO7, and O1); right anterior/RA (F4, F6, F8, FC4, FC6, and FT8); right central/RC (C4, C6, T8, CP4, CP6, and TP8); and right posterior/RP (P4, P6, P8, PO4, PO8, and O2). Average the mean ERP amplitude for each ROI over the electrodes in each region. The Greenhouse–Geisser correction was applied where appropriate. All significant interaction effects were followed by *post-hoc* simple effect comparisons (Zhang et al., 2009). In addition, the 7 anterior frontal electrodes (Fp1, AF3, AF7, Fp2, Fp2, AF4, AF8) were added to the calculation of the P200 component in the homophone task, due to the seemingly significant effect of low-frequency condition on the topographic map.

## Results

### Behavioral Data

**Figure 2:**
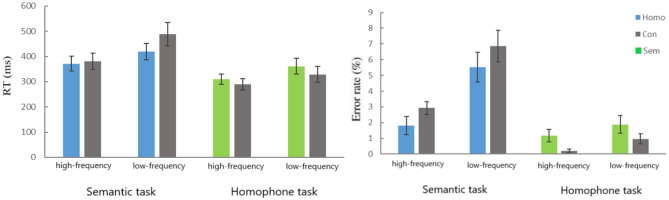
Mean response times (RT) and error rates for the 2 types of trials in both the semantic task and the homophone task (error bars indicate standard error).

In the semantic judgment task, participants need to judge whether the preceding word and the target word are semantically related. In the phonological judgment task, participants need to judge whether the preceding and target word are homophones. The average response times (RT) and error rates of each task are shown in Figure 2. The reaction time and error rate of each task were analyzed using two-factors (relation type and frequency) repeated measurement analysis of variance (Zhang et al., 2009). The behavioral data was recorded from the onset of interface “?” which the participant needs to make a judgment.

The analysis of RT in the semantic judgment task indicated a significant main effect both for Relation Type [Homo vs. Con, 395 vs. 435 ms, *F*_(1,23)_ = 5.856, *P* = 0.024, η^2^_*p*_ = 0.203] and Frequency [High vs. Low, 376 vs. 455 ms, *F*_(1,23)_ = 27.986, *P* < 0.001, η^2^_*p*_ = 0.549], indicating that the RT of low-frequency pairs was longer than that of high-frequency pairs. Additionally, a significant Relation Type × Frequency interaction was found [*F*_(1,23)_ = 7.105, *P* = 0.014, η^2^_*p*_ = 0.236]. *Post-hoc* comparisons showed that response was significantly faster for homophonic pairs than for control pairs for low-frequency pairs [Homo vs. Con, 419 vs. 489 ms, *F*_(1,23)_ = 8.943, *P* = 0.007, η^2^_*p*_ = 0.280], but not for high-frequency pairs [371 vs. 380 ms, *F* < 1].

For the analysis of RT in the homophone judgment task, a main effect for Relation Type [Sem vs. Con, 335 vs. 309 ms, *F*_(1,23)_ = 6.849, *P* = 0.015, η^2^_*p*_ = 0.229] reached significance, revealing that the RT of semantically related pairs was markedly longer than for unrelated pairs. Moreover, there was a main effect for Frequency [High vs. Low, 300 vs. 345 ms, *F*_(1,23)_ = 14.267, *P* = 0.001, η^2^_*p*_ = 0.383], indicating that the RT of low-frequency pairs was significantly longer than that of high-frequency pairs. However, no significant Frequency × Relation Type interaction was found [*F* < 1].

ANOVAs on the error rate data in the semantic judgment task indicated a significant main effect for Frequency [High vs. Low, 2.4 vs. 6.2%, *F*_(1,23)_ = 40.457, *P* = < 0.001, η^2^_*p*_ = 0.638], indicating that the error rate of low-frequency pairs was higher than that of high-frequency pairs. There was a marginally significant main effect for Relation Type [Homo vs. Con, 3.6 vs. 4.8%, *F*_(1,23)_ = 3.894, *P* = 0.061, η^2^_*p*_ = 0.145], revealing that the error rate of phonological pairs was slightly lower than for unrelated pairs. There was no significant Frequency × Relation Type interaction [*F* < 1].

The analysis of error rate data in homophone judgment task revealed a significant main effect for Frequency [High vs. Low, 0.7 vs 1.4%, *F*_(1,23)_ = 5.970, *P* = 0.023, η^2^_*p*_ = 0.206], indicating that the error rate of low-frequency pairs was higher than that of high-frequency pairs. There was a significant main effect for Relation Type [Sem vs. Con, 1.5 vs. 0.6%, *F*_(1,23)_ = 7.126, *P* = 0.014, η^2^_*p*_ = 0.237], revealing that the error rate of semantically related pairs is significantly higher than that of unrelated pairs. There was also no significant Frequency × Relation Type interaction [*F* < 1].

### Electrophysiological Data

**Figure 3:**
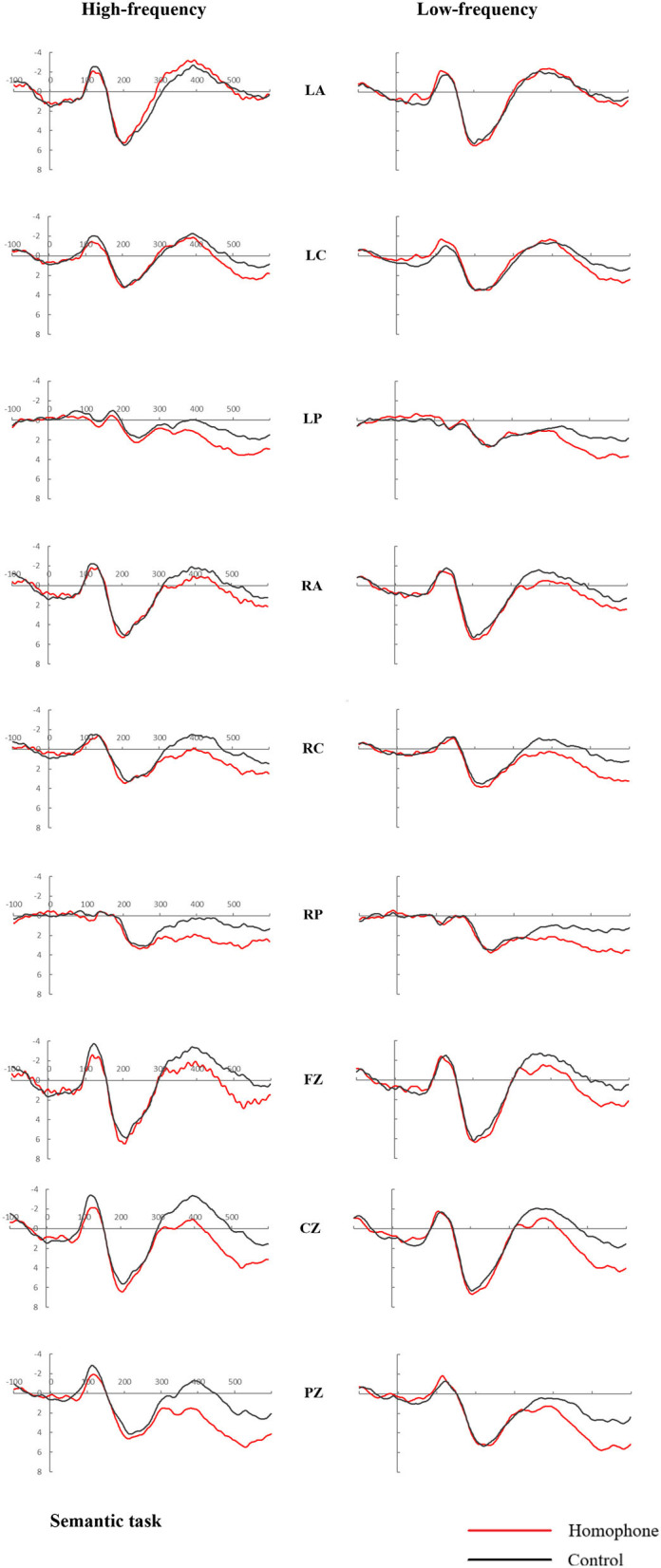
Grand mean event-related potentials in response to target words, from representative electrodes (LA, LC, LP, RA, RC, RP, Fz, Cz, and Pz), for homophonic and control pairs in the semantic task.

**Figure 4:**
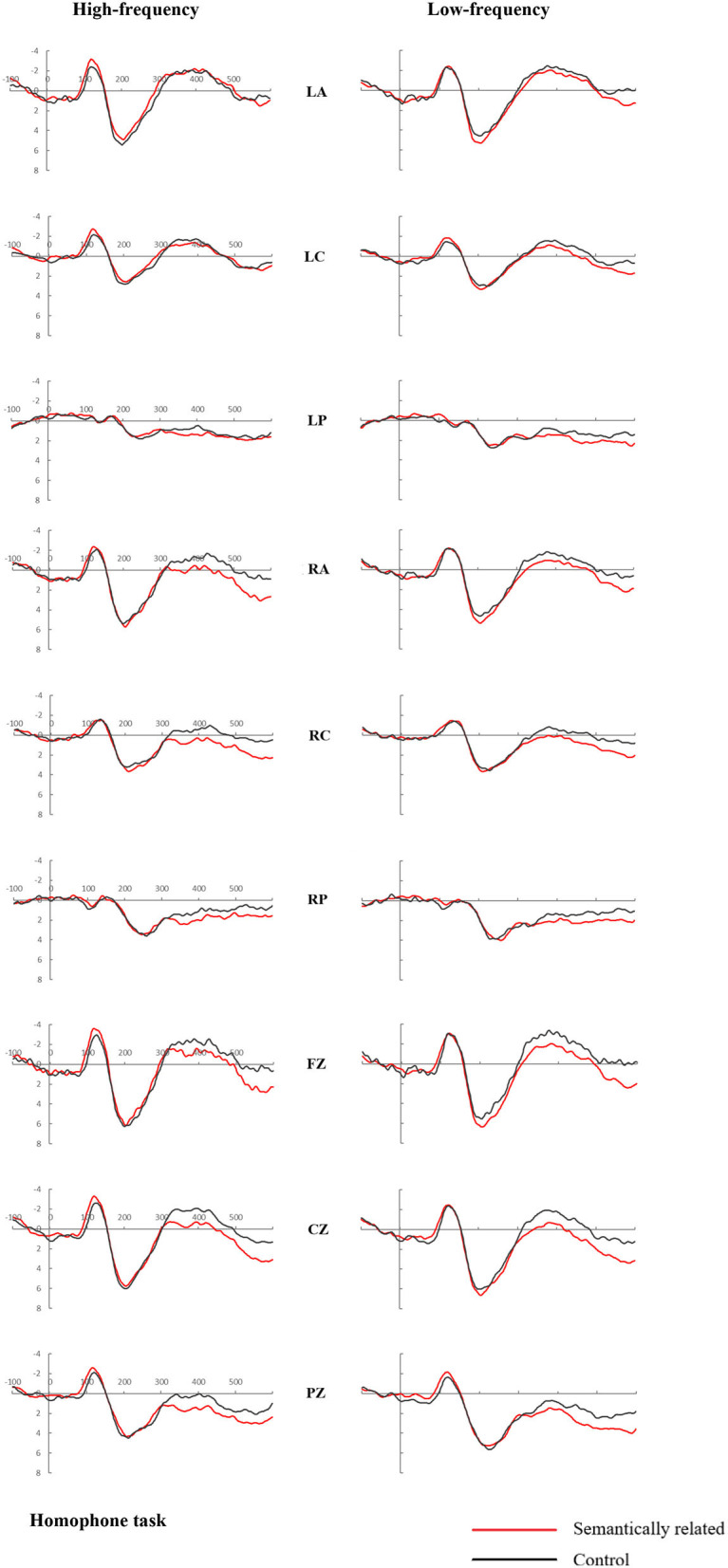
Grand mean event-related potentials in response to target words, from representative electrodes (LA, LC, LP, RA, RC, RP, Fz, Cz, and Pz), for the semantically related and control pairs in the homophone task.

Figures 3, 4 showed the average ERPs produced by the target in all negative tests of the both high and low frequency pairs in the 2 tasks. As shown in Figures 3, 4 below, a positive peak is displayed in the time window after 160–280 ms (P200) stimulation and a negative peak was in the 300–500 ms (N400) time window. We would monitor the two ERP components of P200 and N400 to explore the relative activation time of phonology and semantics (Zhang et al., 2009).

### ERP Results for the Semantic Judgment Task

#### The 160–280-ms Period

For the midline electrodes, there was no significant main effect for Relation Type, or interaction of Frequency × Relation Type (*ps* > 0.1). However, there was a main effect of Frequency [*F*_(1,23)_ = 5.546, *P* = 0.027, η^2^_*p*_ = 0.194], indicating that low-frequency pairs elicited a larger positive waveform than high-frequency pairs. No other significant effect or interaction was found.

At the lateral sites, there was also no significant main effect for Relation Type, or interaction of Frequency × Relation Type (*ps* > 0.1). The overall analysis also revealed a main effect of Frequency [*F*_(1,23)_ = 5.585, *P* = 0.027, η^2^_*p*_ = 0.195], indicating that low-frequency pairs elicited a larger positive waveform than high-frequency pairs. A significant Frequency × Region interaction was found [*F*_(5,115)_ = 3.083, *P* = 0.047, η^2^_*p*_ = 0.118]. Further analyses showed that the effect of Frequency was significant in 2 regions [LC: *F*_(1,23)_ = 10.345, *P* = 0.004, η^2^_*p*_ = 0.310; LP: *F*_(1,23)_ = 18.448, *P* < 0.001, η^2^_*p*_ = 0.445].

#### The 300–500-ms Period

For the midline electrodes, a significant main effect of Relation Type was observed [*F*_(1,23)_ = 27.783, *P* < 0.001, η^2^_*p*_ = 0.547], which showed a smaller negative amplitude for the homophone pairs than for the unrelated condition. Additionally, a significant Relation Type × Electrodes interaction was found [*F*_(2,46)_ = 3.597, *P* = 0.053, η^2^_*p*_ = 0.135]. Further analyses revealed that the effect of Relation Type was significant at all 3 midline electrodes [Fz: *F*_(1,23)_ = 10.406, *P* = 0.004, η^2^_*p*_ = 0.312; Cz: *F*_(1,23)_ = 26.809, *P* < 0.001, η^2^_*p*_ = 0.538; and Pz: *F*_(1,23)_ = 46.063, *P* < 0.001, η^2^_*p*_ = 0.667].

**Figure 5:**
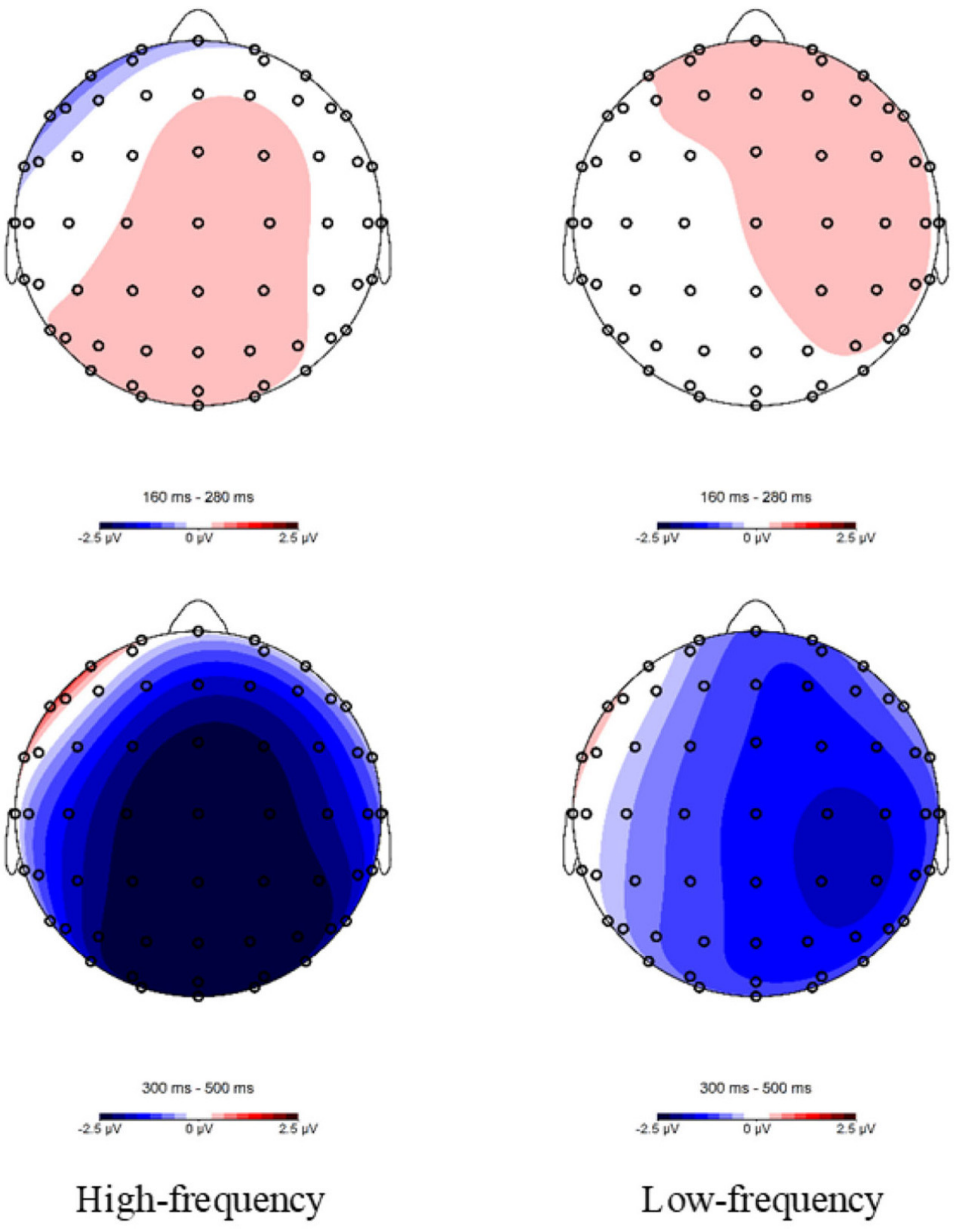
Topographic maps of the different waves in the semantic task for the 160–280 (homophonic minus control) and 300–500-ms (control minus homophonic) epochs following target onset.

At the lateral sites, a significant effect of Relation Type was also observed [*F*_(1,23)_ = 11.862, *P* = 0.002, η^2^_*p*_ = 0.340], indicating that the ERP signal was less negative-going in the homophone condition than in response to unrelated pairs. A marginally significant effect of Frequency was observed [*F*_(1,23)_ = 3.166, *P* = 0.088, η^2^_*p*_ = 0.121]. Additionally, a significant Relation Type × Region interaction was found [*F*_(5,115)_ = 16.969, *P* < 0.001, η^2^_*p*_ = 0.425]. Further analyses revealed that the effect of Relation Type was significant in 4 regions [LP: *F*_(1,23)_ = 17.188, *P* < 0.001, η^2^_*p*_ = 0.428; RA: *F*_(1,23)_ = 8.240, *P* = 0.009, η^2^_*p*_ = 0.264; RC: *F*_(1,24)_ = 24.608, *P* < 0.001, η^2^_*p*_ = 0.517; RP: *F*_(1,23)_ = 44.610, *P* < 0.001, η^2^_*p*_ = 0.660]. The 2 panels of Figure 5 show topographical maps of the differential waves in response to high- and low-frequency pairs; these indicated that the N400 effect of relationship type was observed primarily at midline sites and was larger at lateral sites over the right hemisphere than over the left hemisphere.

### ERP Results for the Homophone Judgment Task

#### The 160–280-ms Period

**Figure 6:**
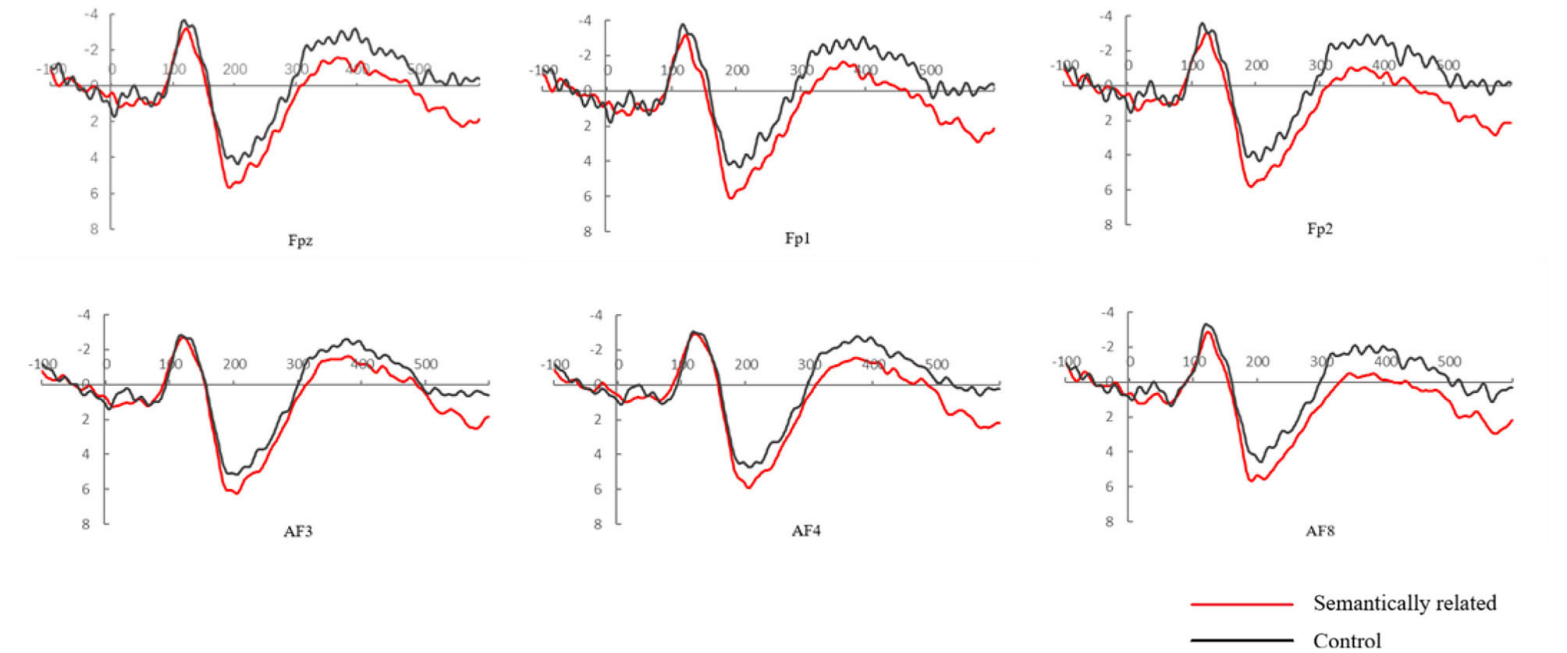
Grand mean event-related potentials in response to target words, from 6 anterior frontal electrodes (Fpz, Fp1, Fp2, AF3, AF4, and AF8), for the semantically related and control pairs of low-frequency in the homophone task.

**Figure 7:**
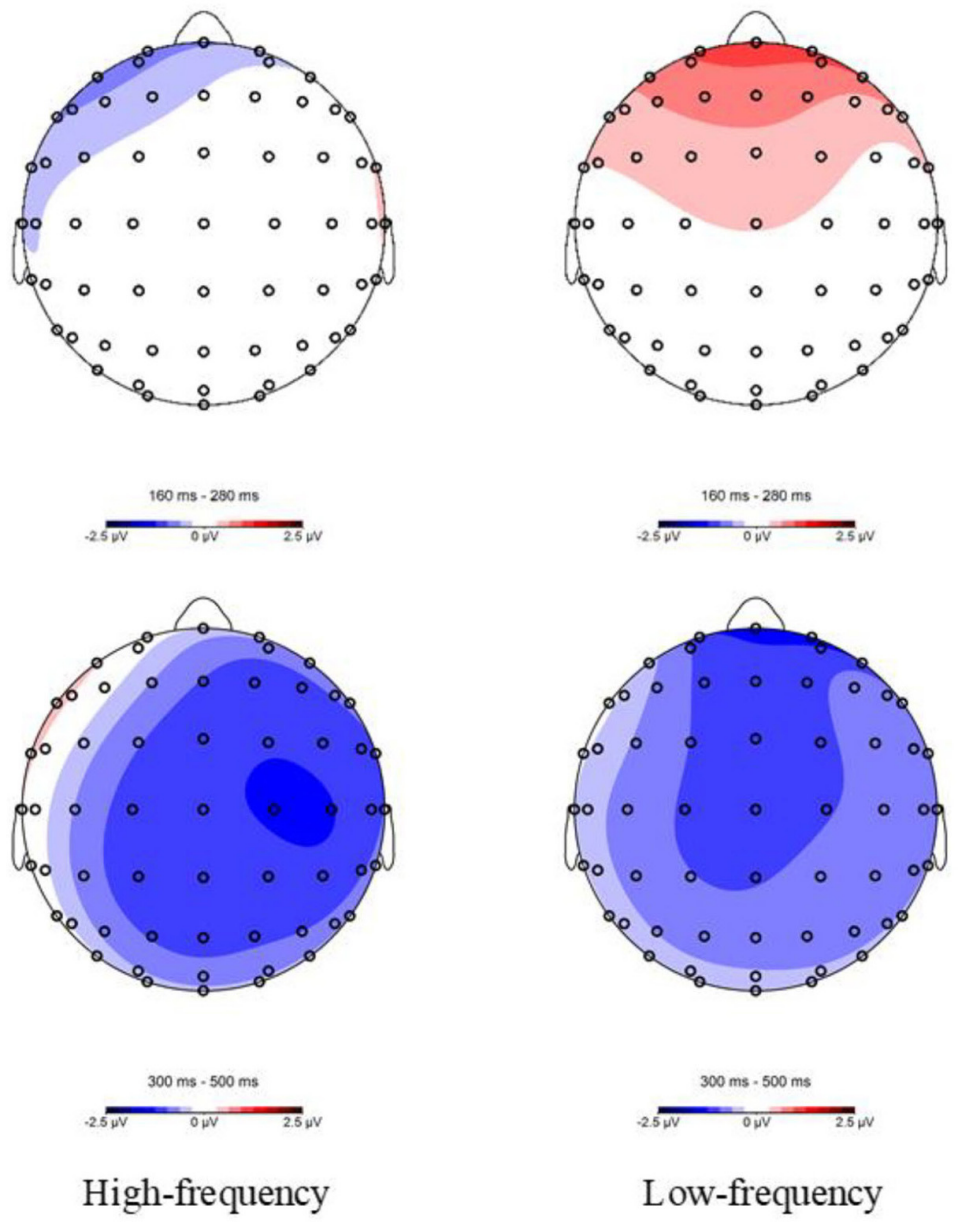
Topographic maps of the difference waves in the homophone task for the 160–280 (semantically related minus control) and 300–500-ms (control minus semantically related) epochs following target onset.

For the anterior frontal electrodes, there was no significant main effect for Relation Type, Frequency (*ps* > .1). A significant Frequency × Relation Type interaction was found [*F*_(1,23)_ = 7.951, *P* = 0.010, η^2^_*p*_ = 0.257]. Further analyses showed that the effect of Relation Type was only significant in low frequency condition (FPz: *P* = 0.055; FP1: *P* = 0.027; FP2: *P* = 0.004; AF3: *P* = 0.060; AF4: *P* = 0.021; AF8: *P* = 0.009), indicating that the ERP signal was more positive-going in the semantically related condition than in the unrelated condition in P200 time window. The following Figure 6 shows in detail the waveform of the 6 anterior frontal electrodes in the low frequency condition, which corresponds to the topographic map in same condition in Figure 7.

For the midline electrodes, there was no significant main effect for Relation Type, Frequency, or their interaction (*ps* > 0.1). A significant Frequency × Electrodes interaction was found [*F*_(2,46)_ = 6.096, *P* = 0.014, η^2^_*p*_ = 0.210]. Further analyses showed that the effect of Frequency was only significant at Pz [*F*_(1,23)_ = 6.564, *P* = 0.017, η^2^_*p*_ = 0.222].

At the lateral sites, there was also no significant effect for Relation Type, Frequency, or their interaction (*F* < 1). Moreover, a significant Frequency × Region interaction was found [*F*_(5,115)_ = 4.363, *P* = 0.016, η^2^_*p*_ = 0.159]. Further analyses showed that the effect of Frequency was only significant in 2 regions [LC: *F*_(1,23)_ = 4.506, *P* = 0.045, η^2^_*p*_ = 0.164; LP: *F*_(1,23)_ = 10.470, *P* = 0.004, η^2^_*p*_ = 0.313].

#### The 300–500-ms Period

For the midline electrodes, a significant main effect of Relation Type was observed [*F*_(1,23)_ = 9.082, *P* = 0.006, η^2^_*p*_ = 0.283], indicating a smaller negative amplitude for the semantically related pairs than for the unrelated condition. Furthermore, a significant Frequency × Electrodes interaction was found [*F*_(2,46)_ = 6.863, *P* = 0.008, η^2^_*p*_ = 0.230]. No other significant effect or interaction was obtained. Further analyses showed that the effect of Frequency was only significant at Pz [*F*_(1,23)_ = 5.526, *P* = 0.028, η^2^_*p*_ = 0.194].

At the lateral sites, a significant effect of Relation Type was also observed [*F*_(1,23)_ = 5.164, *P* = 0.033, η^2^_*p*_ = 0.183], indicating that the ERP signal was less negative-going in the semantically related condition than in the unrelated condition. There was a marginally significant effect of Relation Type × Region interaction [*F*_(5,120)_ = 3.151, *P* = 0.060, η^2^_*p*_ = 0.115]. Further analyses revealed that the effect of Relation Type was significant in 4 regions [LP: *F*_(1,23)_ = 2.991, *P* = 0.097, η^2^_*p*_ = 0.115; RA: *F*_(1,23)_ = 5.795, *P* = 0.024, η^2^_*p*_ = 0.201; RC: *F*_(1,23)_ = 14.063, *P* = 0.001, η^2^_*p*_ = 0.379; RP: *F*_(1,24)_ = 6.845, *P* = 0.015, η^2^_*p*_ = 0.229). The 2 panels of Figure 7 show topographical maps of the differential waves for high- and low-frequency word-pairs (semantically related minus control), indicating that the effect of relationship type was primarily notable at midline sites in the right hemisphere for high-frequency words and in the central-anterior region for low-frequency words.

## Discussion

The main purpose of the present study was to investigate whether phonology was activated and played an important role in reading Chinese two-character compound words of both high and low frequencies. We used a well-established rationale of the interference paradigm of semantic and homophone judgment task, with the ERP technique (Perfetti and Zhang, 1995; Liu et al., 2003; Zhang et al., 2009). For the semantic judgment task, we could compare the behavioral and ERP responses to target words, preceded by homophones and irrelevant words, to reveal when and whether phonology interferes with semantic access. Similarly, for the phonological judgment task, we could compare the behavioral and ERP responses of target words preceded by semantically related and irrelevant words to reveal when and whether semantics interfere with phonological judgement. Then, the time-course of phonological and semantic activation, based on the latency of the relevant ERP components, could be compared and the effect of word frequency on such activation patterns could be examined. The findings from behavioral and ERP data are summarized as follows.

Below, behavioral responses to both high- and low-frequency words in 2 judgment tasks were summarized to infer the relative activation time of phonology and semantics. On the whole, low-frequency words performed worse than high-frequency words in terms of reaction time and error rate both in semantic and phonological judgment tasks. For high-frequency words, in semantic judgment tasks, it was noteworthy that subjects did not make more errors or spend longer times on high-frequency homophone foils than on their corresponding controls. The absence of phonological interference in semantic access in the present study is consistent with earlier research performed mostly on single-character words of high frequency (Chen et al., 1995; Zhou and Marslen-Wilson, 1999, 2000; Zhou et al., 1999). For high-frequency words in the phonological judgment task, subjects made more errors and spent longer on semantic pairs than on unrelated words in homophone judgment task. The interference effect of semantics in phonological tasks is consistent with a previous study on single-character words (Zhang et al., 2009). Overall, for high-frequency words, the behavioral data indicated that phonology does not interfere with access to semantics, but semantics interfere with access to phonology, which suggests that phonological activation would not occur automatically during the semantic access to high-frequency compound words. Nevertheless, for low-frequency words in the semantic judgment task, the behavioral results showed that responses to homophones were faster than to irrelevant word-pairs, seemingly indicating that phonological information may activate earlier and facilitate semantic access. Additionally, for low-frequency words in phonological tasks, the inhibitory effect of semantics contradictorily suggested that semantic information may activate earlier and interfere with phonological processing. Therefore, for low-frequency Chinese words, it was difficult to draw conclusions based only on behavioral results.

ERPs are more functionally decomposable and more trustworthy than behavioral data. Below, the 2 ERP components, P200 and N400, recorded in this study are analyzed in detail. In the semantic judgment task, low-frequency word-pairs were found to induce a larger P200 than high-frequency word-pairs. More importantly, the difference between the homophone pairs and control condition was not found in the P200 time-window, independent of word frequency, replicating the findings for single-character Chinese words of high frequency (Liu et al., 2003; Zhang et al., 2009). In the phonological judgment task, the frequency effect of P200 was also obtained. Note that low-frequency semantic-related pairs released a more positive P200 than the control condition, indicating semantic information plays a role during phonological access. In the literature, P200 usually represents early phonological or orthographic activation in visual word recognition (Barnea and Breznitz, 1998; Sereno et al., 1998; Landi and Perfetti, 2007; Liu et al., 2011). In addition, Sereno et al. (1998) and Barnea and Breznitz (1998) proposed that P200 can reflect vocabulary processing and can be modulated by lexicality, word frequency, and word regularity. At the same time, P200 is widely used to explore the debate about phonological processing in Chinese word recognition. According to the experimental paradigm of previous studies, the paradigms were mostly divided between sentence ending (Meng et al., 2008; Ren et al., 2009; Liu et al., 2011) and word preceding (Liu et al., 2003; Zhang et al., 2009). However, the size of P200 relative to the correct condition (control group) was inconsistent, particularly in the paradigm of sentence ending. These discrepancies may be attributed to contextual factors that are difficult to control in the sentence, since a highly constrained context can influence word-processing (Petten, 1993; Rayner et al., 1998). In contrast, using the paradigm of word priming allows exploration of the role of phonology in word recognition more clearly, as context does not interfere with this. For instance, Zhang et al. (2009) also did not find a priming effect of phonology in the semantic judgment task for high-frequency characters.

In addition to P200, another indispensable ERP component in word recognition is N400, which promises to shed light on the neurological basis of meaning processing (Kutas and Federmeier, 2011; Rabovsky et al., 2018; Huang et al., 2019). The ERP results in this experiment showed that homophones in the semantic judgment task and semantically related words in the phonological judgment task both induced a smaller N400 than did the control words, indicating that phonology and semantics are both activated in the N400 time-window for both high- and low-frequency words. This finding is consistent with some studies on the exploration of Chinese single-character words (Zhang et al., 2009), but also inconsistent with two research results conducted by Chen et al. (2007) and Liu et al. (2003). In the study of Liu et al. (2003), compared with unrelated controls, homophone pairs in the semantic decision task caused the amplitude of the N400 component to decrease, but this phenomenon was not observed in the semantically related word pairs in the phonological decision task. This result may be due to the weak correlation between one-third of the semantically related words in the above study, as Zhang et al. (2009) noted. In addition, it was also surprising that Chen et al. (2007) did not find the N400 component of homophones in the semantic task, which has been strongly proved in other studies (Valdes-Sosa et al., 1993; Barnea and Breznitz, 1998; Zhang et al., 2009).

We will integrate the behavior and ERP data below to infer the semantic access route of Chinese two-character compound words of both high and low frequency more completely. Overall, for high-frequency words, the behavioral data revealed that phonology did not interfere with semantic access, but semantics interfered with phonological processing, indicating that semantics activated earlier than phonology. Complementarily, for high-frequency words, ERP data revealed that phonology and semantics were both activated in the N400 time-window, but did not provide information on their temporal order. Hence, the combined behavioral and ERP results strongly suggested that semantic activation of high-frequency words is earlier or at least no later than that of phonology during recognition of Chinese two-character compound words. Our study is the second ERP experiment to investigate the phonological and semantic processing of Chinese two-character compound words. In the first study, the word frequency factor was not taken into account (Wong et al., 2014). Nevertheless, the conclusions of both studies about Chinese high-frequency words were consistent in concluding that semantic activation may be earlier or at least no later than phonological activation. Nevertheless, a limitation of the study of Wong et al. (2014) was that only the homophone of the first Chinese character or the second Chinese character in the two-character compound words was set. Therefore, a word-level phonological priming effect could not be completely excluded in Chinese two-character compound word recognition. In our experiment, the phonology of the preceding word was the same as that of the target word in the phonology-related condition (on both the first and the second character). Thus, the two ERP experiments on Chinese high-frequency compound words can conclude that semantic activation is at least no later than phonological activation. Note that a previous study (Tse and Yap, 2018) on large-scale two-character compounds regression analysis also opposes the powerful contribution of character phonology in Chinese compound words processing.

No previous ERP study had investigated the role of phonology during semantic access when reading low-frequency Chinese two-character compound words. We found that low-frequency pairs overall induced a larger P200 than did high-frequency word-pairs both in the semantic and the phonological judgment task. Regarding the P200 frequency effect, there may be two explanations. First, this early component may be a carry-over effect of the processing of the precedes. Because our preceding words were only presented for 140 ms, the subjects may still process the precedent when the target words were presented. Thus, the P200 effect may be the carry-over of the precedes' N400 and late positive components. Second, it has been reported that early ERP components are sensitive to lexical frequency (Carreiras et al., 2005; Hauk et al., 2006). So the P200 frequency effect in our experiment may prove a top-down flow of information during visual word recognition.

No differences between the low-frequency homophone pairs and the control condition were observed in the P200 time-window in semantic task. This result indicated that phonology of low-frequency pairs may not be activated in our semantic task around 200 ms post-stimulus onset, which earlier than the semantic activation indexed by the N400 peaking around 400-ms post-stimulus. However, the low-frequency targets preceded by the semantically related words released a larger P200 followed by a smaller N400 than the control condition in the phonological judgment task, which seems to indicate that both phonology and semantic were activated in the P200 time window. This result is not difficult to understand as the phonological activation is expected in the phonological judgment task, but the P200 was triggered by semantic preceding effects, which again seems to indicate that semantic processing is no later than or simultaneous with phonological processing. Although there is no similar study on Chinese two-character compound words of low frequency, there are 2 studies of low-frequency Chinese single-character words (Chen et al., 2007; Zhang et al., 2009). It should be noted that both a larger P200 was induced by the homophone condition in two previous semantic judgment studies, but a reduced N400 was found by Zhang et al. (2009) and an opposite phenomenon—increased N400—was found by Chen et al. (2007), relative to unrelated control pairs. The results of the 2 experiments above may reflect the assumption that phonology was activated during the semantic access of low-frequency Mandarin single-character words, but it may not be suitable for Chinese two-character compound words. A Chinese two-character word is composed of two separate Mandarin characters, and each one usually serves as a morpheme. In recent years, the research on the morphological processing mechanism of Chinese basically confirmed that morphological decomposition is pre-lexical and early morphological processing is sensitive to morphemic meanings (Tsang and Chen, 2013a,b; Tsang and Chen, 2014; Wu et al., 2017). Therefore, the influence of the whole lexical meaning on early morphological processing may reinforce the hypothesis that a top-down flow of information during visual word recognition does exist.

Note that low-frequency homophones in the semantic judgment task induced a smaller N400 than did the control words, indicating that phonology and semantics were both activated in the N400 time-window. Even in phonological judgment tasks that require the retrieval of phonological information, P200, which characterizes phonological processing, was generated due to semantic preceding. Thus, phonological activation for low-frequency words may be no earlier than or simultaneous with semantic processing. Few studies on Chinese word recognition have considered the influence of word frequency. In studies of alphabetic languages, some argue that meanings are always activated by phonological representations (Van Orden, 1987; Frost, 1998). There is a lot of evidence that phonology will be activated in skilled readers when reading low-frequency words, but whether it is suitable for high-frequency words is still unclear (Jared et al., 1999). Experiments conducted by Newman et al. (2012) revealed that phonology continues to play a role in activating word meanings, even for highly practiced words, providing evidence that activation of phonology is not affected by word frequency during the process of English word recognition. The relative regularity of the mapping between orthography and phonology, and hence the efficiency of calculation, may lead to an important role of phonology in the semantic access of alphabetic language (Zhou et al., 1999). In contrast, the relation between orthography and phonology is more arbitrary in Chinese than in alphabetic scripts. Chinese characters with similar orthography usually have different pronunciations, while characters with different orthography may have the same pronunciation. Thus, in Chinese, the contribution of phonology to the activation of meaning may be limited both for high- and low- frequency compound words. However, some studies specifically focus on Chinese phonological effects, including phonological consistency effect (Tse et al., 2017) and the homophone density effect (Chen et al., 2009), tend to be obtained in low frequency words. These findings seem to provide evidence for the role of single-character phonology in recognizing low-frequency two-character compounds. However, phonological consistency is difficult to distinguish from morphemic or character consistency. Therefore, it is not clear whether the effect of phonological consistency is purely phonological or can be partly attributed to 2 competing meanings (Tse and Yap, 2018).

Another explanation for our results may be the interaction between semantics and phonology. The current researches on Chinese word recognition are most based on the hypothesis of feedforward manner, but Carreiras et al. (2014) proposed that the system may be fully interactive, with low-level information flowing bottom-up to the whole lexical information, while simultaneously high-level information flowing top-down to form early word recognition. Two complementary tasks were performed in our study (a semantic and a phonological one). The first one consisted in a semantic judging task which a priori did not require any phonological processing and the second one a phonological matching task which a priori did not require any semantic processing. However, both phonological processing in semantic tasks and semantic processing in phonology tasks were discovered. The time of interaction between the 2 seems to vary with frequency and task. For high-frequency words, only a significantly less negative N400 was found both in the semantic and phonological task, that is, the interaction between semantics and phonology was found only in the N400 time window. However, for low-frequency words, in addition to the N400 effect that appeared in both tasks, a significantly more positive P200 component triggered by semantically related pairs was obtained in phonological task. In other words, for low-frequency words, the interaction between phonology and semantics occurred in the N400 time window in the semantic task, but advanced to the P200 time window in the phonological task. Consequently, high-order lexical–semantic information may constrain phonological-level processing in the P200.

As expounded above, higher-level linguistic information may already exert its influence at 200 ms from stimulus onset. Based on the above assumptions, it is not difficult to understand why low-frequency words released a significantly more positive P200 component than high-frequency words in both tasks. Other studies have also reported that ERP components of about 100 to 200 ms are sensitive to word frequency (Carreiras et al., 2005; Hauk et al., 2006). Since the early ERP components of word recognition (N170, N250) are affected by higher-order word information, Carreiras et al. (2014) supported an interactive activation model for word recognition. In this model, information from low-level visual features flowed bottom-up to the entire lexical representation, while at the same time it can flow from higher-level representations to the bottom (Carreiras et al., 2014). Based on that different languages have different relationships between orthography, phonology, and semantics (or other representations), Frost (2012) also argued that interactive models that allow phonology, morphological and semantic information to work as early as possible can better account for the cross-language differences observed in early word recognition.

The present study of reading Chinese two-character compound-words is the second ERP-based study investigating the role of phonology, but no previous study has investigated both high- and low-frequency words. In addition, unlike the paradigm of the first investigation, a well-established rationale of the interference paradigm was implemented in the current study of semantic and homophone judgment tasks. All in all, we showed two fundamental points both in high- and low-frequency words: (i) a semantic processing in phonological tasks and (ii) a phonological processing in semantic tasks. Therefore, representations such as semantics and phonology may be interactive and higher-order linguistic representations may modulate early processing.

## Data Availability Statement

The raw data supporting the conclusions of this article will be made available by the authors, without undue reservation.

## Ethics Statement

The studies involving human participants were reviewed and approved by the ethics committee of the School of Psychology at Tsinghua University. The patients/participants provided their written informed consent to participate in this study. Written informed consent was obtained from the individual(s) for the publication of any potentially identifiable images or data included in this article.

## Author Contributions

YW, MJ, and YH: conceived, designed the experiments, data collection, and analyzed the data. YW and YH: performed the experiments. YW, MJ, YH, and PQ: contributed wrote the paper. All authors reviewed the manuscript.

## Conflict of Interest

The authors declare that the research was conducted in the absence of any commercial or financial relationships that could be construed as a potential conflict of interest.
