# Supplementary material for: An ERP Study on the Role of Phonological Processing in Reading Two-Character Compound Chinese Words of High and Low Frequency
Source: Front Psychol. 2021 Feb 25;12:637238. doi: 10.3389/fpsyg.2021.637238 (PMC7947322; doi:10.3389/fpsyg.2021.637238)
Supplement: Supplementary file 1 [file Data_Sheet_1.PDF]

APPENDIX

The first 60 rows of words form the high frequency group while the rest form the low frequency group.

	**Phonological** precede	**Semantic** precede	**Control** precede	**Target**
1	精力 jing(1)li(4)	体验 ti(3)yan(4)	政治 zheng(4)zhi(4)	经历 jing(1)li(4)
2	形成 xing(2)cheng(2)	路线 lu(4)xian(4)	要求 yao(1)qiu(2)	行程 xing(2)cheng(2)
3	店员 dian(4)yuan(2)	插座 cha(1)zuo(4)	非常 fei(1)chang(2)	电源 dian(4)yuan(2)
4	消瘦 xiao(1)shou(4)	市场 shi(4)chang(3)	母亲 mu(3)qin(1)	销售 xiao(1)shou(4)
5	进士 jin(4)shi(4)	眼镜 yan(3)jing(4)	活动 huo(2)dong(4)	近视 jin(4)shi(4)
6	失声 shi(1)sheng(1)	学校 xue(2)xiao(4)	汽车qi(4)che(1)	师生 shi(1)sheng(1)
7	报时 bao(4)shi(2)	肥胖 fei(2)pang(4)	中间 zhong(1)jian(1)	暴食 bao(4)shi(2)
8	闻名 wen(2)ming(2)	礼貌 li(3)mao(4)	计算 ji(4)suan(4)	文明 wen(2)ming(2)
9	骑士 qi(2)shi(4)	小看 xiao(3)kan(4)	立刻 li(4)ke(4)	歧视 qi(2)shi(4)
10	同化 tong(2)hua(4)	寓言 yu(4)yan(2)	批评 pi(1)ping(2)	童话 tong(2)hua(4)
11	密封 mi(4)feng(4)	昆虫 kun(1)chong(2)	身体 sheng(1)ti(3)	蜜蜂 mi(4)feng(4)
12	例证 li(4)zheng(4)	笔直 bi(3)zhi(2)	严重 yan(2)zhong(4)	立正 li(4)zheng(4）
13	冲击 chong(1)ji(1)	吃饱 chi(1)bao(3)	文学 wen(2)xue(2)	充饥 chong(1)ji(1)
14	遗弃 yi(2)qi(4)	设备 she(4)bei(4)	道理 dao(4)li(3)	仪器 yi(2)qi(4)
15	饱食 bao(3)shi(2)	钻戒 zuan(4)jie(4)	矛盾 mao(2)dun(4)	宝石 bao(3)shi(2)
16	升值 sheng(1)zhi(2)	怀孕 huai(2)yun(4)	旁边 pang(2)bian(1)	生殖 sheng(1)zhi(2)
17	经验 jing(1)yan(4)	漂亮 piao(4)liang(0)	明天 ming(2)tian(1)	惊艳 jing(1)yan(4
18	异议 yi(4)yi(4)	价值 jia(4)zhi(2)	服务 fu(2)wu(4)	意义 yi(4)yi(4)
19	只是 zhi(3)shi(4)	命令 ming(4)ling(4)	石油 shi(2)you(2)	指示 zhi(3)shi(4)
20	视线 shi(4)xian(4)	地区 di(4)qu(1)	热情 re(4)qing(2)	市县 shi(4)xian(4)
21	石狮 shi(2)shi(1)	执行 zhi(2)xing(2)	电影 dian(4)ying(3)	实施 shi(2)shi(1)
22	芝士 zhi(1)shi(4)	学问 xue(2)wen(4)	门口 men(2)kou(4)	知识 zhi(1)shi(4)
23	现成 xian(4)cheng(2)	乡村 xiang(1)cun(1)	首先 shou(3)xian(1)	县城 xian(4)cheng(2)
24	行事 xing(2)shi(4)	局面 ju(2)mian(4)	材料 cai(2)liao(4)	形势 xing(2)shi(4
25	立意 li(4)yi(4)	好处 hao(3)chu(4)	物质 wu(4)zhi(4)	利益 li(4)yi(4)
26	纪实 ji(4)shi(2)	秒表 miao(3)biao(3)	平原 ping(2)yuan(2)	计时 ji(4)shi(2)
27	起夜 qi(3)ye(4)	公司 gong(1)si(1)	内容 nei(4)rong(2)	企业 qi(3)ye(4
28	时务 shi(2)wu(4)	包子bao(1)zi(0)	成立 cheng(2)li(4)	食物 shi(2)wu(4)
29	旺季 wang(4)ji(4)	回忆 hui(2)yi(4)	道理 dao(4)li(3)	忘记 wang(4)ji(4)
30	升涨 sheng(1)zhang(3)	成熟 cheng(2)shu(2)	责任 ze(2)ren(4)	生长 sheng(1)zhang(3)
31	示例 shi(4)li(4)	眼睛 yan(3)jing(0)	小时 xiao(3)shi(2)	视力 shi(4)li(4)
32	事迹 shi(4)ji(4)	时代 shi(2)dai(4)	介绍 jie(4)shao(4)	世纪 shi(4)ji(4)
33	职务 zhi(2)wu(4)	树木 shu(4)mu(4)	互相 hu(4)xiang(1)	植物 zhi(2)wu(4)
34	河里 he(2)li(3)	过分 guo(4)fen(4)	到处 dao(4)chu(4)	合理 he(2)li(3)
35	紧紧 jin(3)jin(3)	只有 zhi(3)you(3)	自由 zi(4)you(2)	仅仅 jin(3)jin(3)
36	工艺 gong(1)yi(4)	爱心 ai(4)xin(1)	报告 bao(4)gao(4)	公益 gong(1)yi(4)
37	织布 zhi(1)bu(4)	党委 dang(3)wei(3)	简单 jian(3)dan(1)	支部 zhi(1)bu(4)
38	集齐 ji(2)qi(2)	特别 te(4)bie(2)	昨天 zuo(2)tian(1)	极其 ji(2)qi(2
39	一栋 yi(2)dong(4)	联通 lian(2)tong(1)	哲学 zhe(2)xue(2)	移动 yi(2)dong(4
40	画功 hua(4)gong(1)	石油 shi(2)you(2)	天气 tian(1)qi(4)	化工 hua(4)gong(1)
41	晶莹 jing(1)ying(2)	销售 xiao(1)shou(4)	耳朵 er(3)duo(0)	经营 jing(1)ying(2)
42	声誉 sheng(1)yu(4)	怀孕 huai(2)yun(4)	商品 shang(1)pin(3)	生育 sheng(1)yu(4)
43	先验 xian(1)yan(4)	暗淡 an(4)dan(4)	处理 chu(3)li(3)	鲜艳 xian(1)yan(4)

44	预示 yu(4)shi(4)	厕所 ce(4)suo(3)	具体 ju(4)ti(3)	浴室 yu(4)shi(4)
45	鸡冠 ji(1)guan(1)	单位 dan(1)wei(4)	最近 zui(4)jin(4)	机关 ji(1)guan(1)
46	奇异 qi(2)yi(4)	明确 ming(2)que(4)	宣传 xuan(1)chuan(2)	歧义 qi(2)yi(4)
47	减值 jian(3)zhi(2)	几乎 ji(1)hu(1)	资料 zi(1)liao(4)	简直 jian(3)zhi(2)
48	笔试 bi(3)shi(4)	小看 xiao(3)kan(4)	成绩 cheng(2)ji(4)	鄙视 bi(3)shi(4)
49	前程 qian(2)cheng(2)	真心 zhen(1)xin(1)	原因 yuan(2)yin(1)	虔诚 qian(2)cheng(2)
50	祝愿 zhu(4)yuan(4)	生病 sheng(1)bing(4)	同学 tong(2)xue(2)	住院 zhu(4)yuan(4)
51	加剧 jia(1)ju(4)	沙发 sha(1)fa(1)	目的 mu(4)di(3)	家具 jia(1)ju(4)
52	劣势 lie(4)shi(4)	牺牲 xi(1)sheng(4)	提供 ti(2)gong(1)	烈士 lie(4)shi(4)
53	夹心 jia(1)xin(1)	升职 sheng(1)zhi(2)	例如 li(4)ru(2)	加薪 jia(1)xin(1)
54	揭示 jie(1)shi(4)	繁华 fan(2)hua(2)	报纸 bao(4)zhi(3)	街市 jie(1)shi(4)
55	连结 lian(2)jie(2)	朴素 pu(3)su(4)	人口 ren(2)kou(3)	廉洁 lian(2)jie(2)
56	实证 shi(2)zheng(4)	热点 re(4)dian(3)	论文 lun(4)wen(2)	时政 shi(2)zheng(4)
57	现世 xian(4)shi(4)	地区 di(4)qu(1)	帽子 mao(4)zi(0)	县市 xian(4)shi(4)
58	实心 shi(2)xin(1)	兼职 jian(1)zhi(2)	似乎 si(4)hu(2)	时薪 shi(2)xin(1)
59	明丽 ming(2)li(4)	声望 sheng(1)wang(4)	实验 shi(2)yan(4)	名利 ming(2)li(4)
60	便意 bian(4)yi(4)	遗传 yi(2)chuan(2)	商品 shang(1)pin(3)	变异 bian(4)yi(4)
61	耗竭 hao(4)jie(2)	皎白 jiao(3)bai(2)	粗疏 cu(1)shu(1)	皓洁 hao(4)jie(2)
62	中数 zhong(1)shu(4)	孝悌 xiao(4)ti(4)	茶坊 cha(2)fang(1)	忠恕 zhong(1)shu(4)
63	颌骨 he(2)gu(3)	黍米 shu(3)mi(3)	权柄 quan(2)bing(3)	禾谷 he(2)gu(3)
64	肃立 su(4)li(4)	姣好 jiao(1)hao(3)	讳言 hui(4)yan(2)	素丽 su(4)li(4)
65	货量 huo(4)liang(4)	晦暗 hui(4)an(4)	酣梦 han(1)meng(4)	豁亮 huo(4)liang(4
66	参试 can(1)shi(4)	饭厅 fan(4)ting(1)	怅然 chang(4)ran(2)	餐室 can(1)shi(4)
67	风蚀 feng(1)shi(2)	充盈 chong(1)ying(2)	芦蒿 lu(2)hao(1)	丰实 feng(1)shi(2)
68	附议 fu(4)yi(4)	田税 tian(2)shui(4)	镂空 lou(2)kong(1)	赋役 fu(4)yi(4)
69	丰益 feng(1)yi(4)	倜傥 ti(4)tang(3)	莴苣 wo(1)ju(4)	风逸 feng(1)yi(4)
70	古稀 gu(3)xi(1)	红利 hong(2)li(4)	辞章 ci(2)zhang(1)	股息 gu(3)xi(1)
71	榆钱 yu(2)qian(2)	湘鄂 xiang(1)e(4)	筹措 chou(2)cuo(4)	渝黔 yu(2)qian(2)
72	书立 shu(1)li(4)	婉约 wan(3)yue(1)	营私 ying(2)si(1)	淑丽 shu(1)li(4)
73	败叶 bai(4)ye(4)	探访 tan(4)fang(3)	媲美 pi(4)mei(3)	拜谒 bai(4)ye(4)
74	霉雨 mei(2)yu(3)	笑靥 xiao(4)ye(4)	凌驾 ling(2)jia(4)	眉宇 mei(2)yu(3)
75	凌宇 ling(2)yu(3)	监牢 jian(1)lao(2)	倒戈 dao(3)ge(1)	图圄 ling(2)yu(3)
76	岂是 qi(3)shi(4)	赌咒 du(3)zhou(4)	缔约 di(4)yue(1)	起誓 qi(3)shi(4)
77	轻拂 qing(1)fu(2)	安闲 an(1)xian(2)	盐碱 yan(2)jian(3)	清福 qing(1)fu(2)
78	示警 shi(4)jing(3)	流俗 liu(2)su(2)	斟酌 zhen(1)zhuo(2)	市井 shi(4)jing(3)
79	鲫鱼 ji(4)yu(2)	妄图 wang(4)tu(2)	猖獗 chang(1)jue(2)	觊觎 ji(4)yu(2)
80	夹击 jia(1)ji(1)	野雉 ye(3)zhi(4)	宽宥 kuan(1)you(4)	家鸡 jia(1)ji(1)
81	球市 qiu(2)shi(4)	监禁 jian(1)jin(4)	甘霖 gan(1)lin(2)	囚室 qiu(2)shi(4)
82	倾心 qing(1)xin(1)	怡然 yi(2)ran(2)	遁去 dun(4)qu(4)	清馨 qing(1)xin(1)
83	幼鱼 you(4)yu(2)	拘泥 ju(1)ni(2)	盘踞 pan(2)ju(4)	囿于 you(4)yu(2)
84	电势 dian(4)shi(4)	遴选 lin(2)xuan(3)	船舷 chuan(2)xian(2)	殿试 dian(4)shi(4)
85	带劲 dai(4)jin(4)	泯灭 min(3)mie(4)	接洽 jia(1)qiao(4)	殆尽 dai(4)jin(4)
86	覆合 fu(4)he(2)	稽查 ji(1)cha(2)	胡琴 hu(2)qin(2)	复核 fu(4)he(2)
87	飞鸿 fei(1)hong(2)	黛绿 dai(4)lv(4)	督率 du(1)shuai(4)	绯红 fei(1)hong(2)
88	俯仰 fu(2)yang(3)	监护 jian(1)hu(4)	革履 ge(2)lv(3)	扶养 fu(2)yang(3)
89	归一 gui(1)yi(1)	加持 jia(1)chi(2)	湮没 yan(1)mo(4)	皈依 gui(1)yi(1)
90	倾卸 qing(1)xie(4)	对虾 dui(4)xia(1)	官宦 guan(1)huan(4)	青蟹 qing(1)xie(4)
91	绯夜 fei(1)ye(4)	内封 fei(4)feng(1)	俸禄 feng(4)lu(4)	扉页 fei(1)ye(4)
92	溶汇 rong(2)hui(4)	通透 tong(1)tou(4)	洗濯 xi(3)di(2)	融会 rong(2)hui(4)
93	倾销 qing(1)xiao(1)	碧空 bi(4)kong(1)	狐疑 hu(2)yi(2)	青霄 qing(1)xiao(1)
94	逆市 ni(4)shi(4)	冷观 leng(3)guan(1)	伏案 fu(2)an(4)	睨视 ni(4)shi(4)

95	目眶 mu(4)kuang(4)	铅锌 qian(1)xin(1)	沟壕 gou(1)hao(2)	钼矿 mu(4)kuang(4）
96	明慧 ming(2)hui(4)	避忌 bi(4)ji(4)	孤孀 gu(1)shuang(1)	名讳 ming(2)hui(4)
97	开市 kai(1)shi(4)	擦抹 ca(1)mo(3)	歉收 qian(4)shou(1)	揩拭 kai(1)shj(4)
98	经纪 jing(1)ji(4)	惶恐 huang(2)kong(3)	攀越 pan(1)yue(4)	惊悸 jing(1)ji(4)
99	后裔 hou(4)yi(4)	盛情 sheng(4)qing(2)	桅杆 wei(2)gan(1)	厚意 hou(4)yi(4)
100	丙烯 bing(3)xi(1)	发怵 fa(1)chu(4)	凝脂 ning(2)zhi(1)	屏息 bing(3)xi(1)
101	结利 jie(2)li(4)	零丁 ling(2)ding(1)	抹煞 mo(3)sha(4)	孑立 jie(2)li(4)
102	休市 xiu(1)shi(4)	神职 shen(2)zhi(2)	暮春 mu(4)chun(1)	修士 xiu(1)shi(4)
103	监查 jian(1)cha(2)	冲泡 chong(1)pao(4)	脉冲 mai(4)chong(1)	煎茶 jian(1)cha(2)
104	进制 jin(4)zhi(4)	酣畅 han(1)chang(4)	茅亭 mao(2)ting(2)	尽致 jin(4)zhi(4)
105	顾及 gu(4)ji(2)	顽症 wan(2)zheng(4)	蛮劲 man(2)jin(4)	痼疾 gu(4)ji(2)
106	休憩 xiu(1)qi(4)	拆卸 chai(1)xie(4)	寥落 liao(2)luo(4)	修葺 xiu(1)qi(4)
107	经售 jing(1)shou(4)	肥硕 fei(2)shuo(4)	劳顿 lao(2)dun(4)	精瘦 jing(1)shou(4)
108	胶质 jiao(1)zhi(4)	泰然 tai(4)ran(2)	知悉 zhi(1)xi(1)	焦炙 jiao(1)zhi(4)
109	宪制 xian(4)zhi(4)	考据 kao(3)ju(4)	淫威 yin(2)wei(1)	县志 xian(4)zhi(4)
110	浮世 fu(2)shi(4)	擦除 ca(1)chu(2)	鼓噪 gu(3)zao(4)	拂拭 fu(2)shi(4)
111	自缢 zi(4)yi(4)	大肆 da(4)si(4)	皂荚 zao(4)jia(2)	恣意 zi(4)yi(4)
112	甄姬 zhen(1)ji(1)	搜捕 sou(1)bu(3)	扪心 men(2)xin(1)	侦缉 zen(1)ji(1)
113	直译 zhi(1)yi(4)	迟疑 chi(2)yi(2)	冗杂 rong(3)za(2)	执意 zhi(2)yi(4)
114	时移 shi(2)yi(2)	拣获 jian(3)huo(4)	疏浚 shu(1)jun(4)	拾遗 shi(2)yi(2)
115	语意 yu(3)yi(4)	爪牙 zhao(3)ya(2)	拂晓 fu(2)xiao(3)	羽翼 yu(3)yi(4)
116	微贷 wei(1)dai(4)	告急 gao(4)ji(2)	田埂 tian(2)geng(3)	危殆 wei(1)dai(4)
117	研习 yan(2)xi(2)	秉承 bing(3)cheng(2)	梦呓 meng(4)yi(4)	沿袭 yan(2)xi(2)
118	雾面 wu(4)mian(4)	接见 jie(1)jian(4)	羸弱 lei(2)ruo(4)	晤面 wu(4)mian(4)
119	集聚 ji(2)ju(4)	紧迫 jin(3)po(4)	顺遂 shui(4)sui(4)	急遽 ji(2)ju(4)
120	池浴 chi(2)yu(4)	名扬 ming(2)yang(2)	赫然 he(4)ran(2)	驰誉 chi(2)yu(4)
